# Extracellular Vesicles from the Protozoa *Acanthamoeba castellanii*: Their Role in Pathogenesis, Environmental Adaptation and Potential Applications

**DOI:** 10.3390/bioengineering6010013

**Published:** 2019-02-01

**Authors:** Diego de Souza Gonçalves, Marina da Silva Ferreira, Allan J. Guimarães

**Affiliations:** 1 Departamento de Microbiologia e Parasitologia, Instituto Biomédico, Universidade Federal Fluminense, Rio de Janeiro 24210-130, Brazil; diegosg@id.uff.br; 2 Departamento de Imunologia, Instituto de Microbiologia Professor Paulo de Góes, Universidade Federal do Rio de Janeiro, Rio de Janeiro 21941-970, Brazil; marinaferreira83@gmail.com

**Keywords:** *Acanthamoeba castellanii*, pathogenesis, adaptation, applications, extracellular vesicles

## Abstract

Extracellular vesicles (EVs) are membranous compartments of distinct cellular origin and biogenesis, displaying different sizes and include exosomes, microvesicles, and apoptotic bodies. The EVs have been described in almost every living organism, from simple unicellular to higher evolutionary scale multicellular organisms, such as mammals. Several functions have been attributed to these structures, including roles in energy acquisition, cell-to-cell communication, gene expression modulation and pathogenesis. In this review, we described several aspects of the recently characterized EVs of the protozoa *Acanthamoeba castellanii,* a free-living amoeba (FLA) of emerging epidemiological importance, and compare their features to other parasites’ EVs. These *A. castellanii* EVs are comprised of small microvesicles and exosomes and carry a wide range of molecules involved in many biological processes like cell signaling, carbohydrate metabolism and proteolytic activity, such as kinases, glucanases, and proteases, respectively. Several biomedical applications of these EVs have been proposed lately, including their use in vaccination, biofuel production, and the pharmaceutical industry, such as platforms for drug delivery.

## 1. Introduction

### 1.1. Free Living Amoebae (FLA)

Free-living amoebas are ubiquitous protozoa widely distributed in various ecological niches being found in soils and lakes, as well as man-made sources such as drinking water reservoirs, swimming pools, cooling towers, and ophthalmic products, often associated with other organisms [1]. The literature has described the isolation of FLAs from various invertebrate and vertebrate superior organisms, including fish, reptiles, amphibians, birds, and mammals. In humans, amoebae infections can range from eye infections and keratitis to infections of higher occurrence, such as encephalitis in the central nervous system (CNS) and are caused by FLAs belonging to the genus *Acanthamoeba* and *Naegleria*, *Balamuthia*, and less frequently, *Sappinia* [2,3,4,5,6,7,8]. Parasitic FLAs could also be commonly found in other sites in association with microbial biofilms, which in turn can be used as a source of nutrients by FLAs or as a protective transient environment for their cysts, especially under unfavorable circumstances to the survival of the trophozoites phase [9,10,11]. 

A major problem faced with FLAs is the contamination of water treatment plants. The cysts are extremely resistant to adverse physical conditions and to treatment with the most common disinfectants, making their elimination virtually impossible, thus composing one of the most abundant contaminant populations following water treatment. When associated with biofilms in these environments, these FLAs are very difficult to eliminate, by becoming less susceptible to chlorine-based disinfectants, thus promoting an increase in bacterial biomass density, which could in turn further protect their cysts [12,13,14].

Recent data addressing water treatment plants in Spain have shown that 90% of the treated water had *Acanthamoeba* spp. as residual contaminants [5]. Coincidentally, in Brazil, the most comprehensive study done in Laguna dos Patos, Rio Grande do Sul, detected the presence of this FLA in 91.7% of the stored water samples evaluated [4]. However, the real number of infections caused by FLAs worldwide is unknown, being classified as agents of neglected diseases. In parallel, the number of death reports caused by FLAs in public health systems worldwide is very scarce; in a study conducted in the United States, the number of reported/documented deaths after patient’s contact with FLA contaminated water averaged only four deaths per year, as notified to the CDC between 2003 and 2009 [15]. Therefore, due to the emerging numbers of FLAs detection from several environmental sources, more effective methodologies for the assessment of FLA contamination and treatment of water reservoirs are extremely necessary to ensure the full healthcare for society and the distribution of an even more salutary water, once this substance is an essential and irreplaceable resource for life.

Another emerging problem regarding FLAs, that has recently been gaining close attention is the presence of endosymbiont pathogens within these FLAs; not only are amoebae themselves a risk to public health, they are also potential reservoirs of epidemiologically important human pathogens, such as bacteria, viruses and endemic fungi [16,17,18,19,20]. Therefore, more in-depth studies of the interactions of FLAs with their environment, including classes of pathogens that inhabit the same FLAs niches, would be a major advance towards the understanding of environmental relations between different organisms and the control of microbial populations.

The importance of FLAs as environmental host is easy to understand in the case of host obligate pathogens. However, when it comes to free-living or facultative pathogens, such as fungi, neither do they require a specific host for survival nor undergo selective pressure with respect to the cause of damage to their potential hosts; in turn, host death often leads to the return of these pathogens to the environment. These concepts generate fundamental questions regarding the potential of soil microorganisms to become virulent and the origin of virulence of facultative pathogens [16,17,18,19,20]. Studies focusing on the interaction between environmental microorganisms such as fungi, bacteria, and viruses with FLAs as a host suggest that various mechanisms by which virulence may emerge or be selected in these accidental hosts, as most of these pathogens are not obligate intracellular [16,18,21,22,23].

### 1.2. FLAs and the Genus Acanthamoeba

*Acanthamoeba* are among the most prevalent environmental protozoa and one of the most widely studied in the laboratory. Several molecular biology techniques have been proposed for the genotyping of the *Acanthamoeba*; however, the 18S rRNA sequencing has been able to classify this genus in at least 20 distinct genotypes (designated from T1–T20) [5,18]. 

The first reports of FLAs belonging to the genus *Acanthamoeba* as the etiological agents of human disease dated from the early 1970s [24,25]. These reports included granulomatous amebic encephalitis (GAE), a fatal disease of the central nervous system (CNS) and amebic keratitis, (AK), a painful sight-threatening disease of the eyes and other skin infections [9]. In addition, human cases of amebic encephalitis were reported soon thereafter in Australia, Europe, Africa, South America, and the United States. Thus, *Acanthamoeba* has been termed amphizoic organisms since they could exist both as free-living amebae and as parasitic pathogens. 

*Acanthamoeba* spp. can act as opportunistic pathogens or as non-opportunistic pathogens. *Acanthamoeba* spp. has also been associated to cutaneous lesions and sinusitis in AIDS patients and other immunocompromised individuals. In GAE, a chronic and progressive CNS infection, which may also involve the lungs, several species of *Acanthamoeba* associated with the disease were found in laboratorial diagnosis. GAE is, in general, associated with immunocompromised individuals, such as malignancies, systemic lupus erythematosus, diabetes, renal failure, cirrhosis, tuberculosis, skin ulcers, human immunodeficiency virus (HIV) infection, or Hodgkin’s disease [9]. Besides that, cases of GAE by *Acanthamoeba* have been found in immunocompetent children and adults [26,27,28,29,30]. The infection occurs by inhalation of amebae through the nasal passages and lungs or the introduction through skin lesions as gateways [29].

The AK is a painful progressive sight-threatening corneal disease and several species of *Acanthamoeba*, including *A. castellanii*, *A. polyphaga*, *A. hatchetti*, *A. culbertsoni*, *A. rhysodes*, *A. griffini*, *A. quina*, and *A. lugdunensis*, have been reported to cause this clinical presentation [31,32]. The individuals affected by AK generally are immunocompetent; however, these individuals do not develop protective immunity, and reinfection can potentially occur [33]. The epidemic of AK in the 1980s was associated to the increased use and poor hygiene of contact lenses [34]. 

Within the *Acanthamoeba* genus, the *A. castellanii* has been the most studied species; besides all the aforementioned human body sites this species could be found, the *A. castellanii* has also been described in pulmonary secretions, maxillary sinuses and stool samples [35]. *A. castellanii* potential and environmental importance as host and bearer of amoeba resistant microorganisms (ARMs), including bacteria, viruses, or fungi, has been acknowledged [18]. These ARMs include potential pathogens, which could be carried within the *A. castellanii* during superior infection to a superior host, through a co-infection mechanism known as Trojan horse [18,36,37,38].

**Figure 1:**
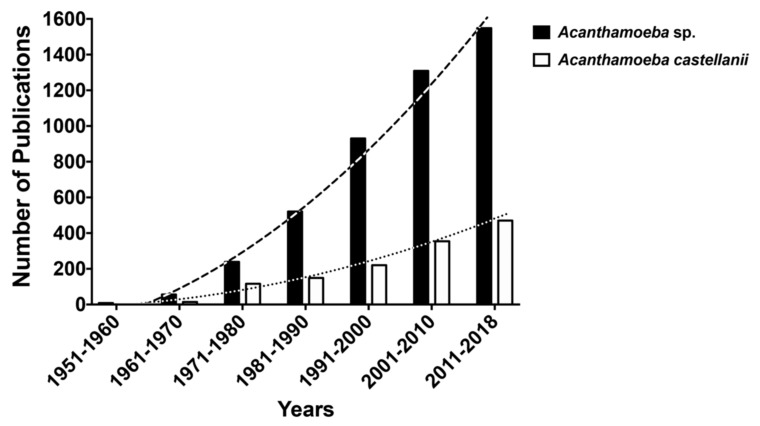
Number of reports in the PubMed database search (www.ncbi. nlm.nih.gov/) within the genus *Acanthamoeba* (4611 publications, dark bars) and specifically *Acanthamoeba castellanii* (1323 publications, white bars), by the month of November 2018.

Over the last 60 years, the number of reports in the PubMed database search (www.ncbi. nlm.nih.gov/) within the genus *Acanthamoeba* includes 4611 publications, by the month of November 2018; when restricting specifically to the *A. castellanii*, the search retrieves 1323 articles (Figure 1). Despite the lower number of publications compared to other protozoa, such as *Trypanosomatids*, publications on *A. castellanii* have been steadily growing over the past 10 years, characterizing an emerging pathogen.

## 2. Extracellular Vesicles (EVs) and Their Role in Microorganism Adaptation

Extracellular vesicles (EVs) are small structures composed of an enclosing lipid bilayer, which possesses a wide variety of constituents depending on the cellular origin, including proteins, enzymes, mRNA, micro RNAs and lipids, which can participate in both the regulation of their surrounding environment, including amoebal population density and regulation of symbionts’ gene expression and may also play a key role during their infection process to a host [39,40].

Based on the size, biogenesis and cellular origin of the molecules that make up the vesicle, they can be classified into different types such as exosomes, microvesicles (MVs) and apoptotic bodies [40,41]. The exosomes are spheres of size ranging from 30 to 100 nm and are commonly formed within the endolysosomal channel, in membranous compartments called multivesicular bodies (MVBs), where they are later addressed to degradative activity in lysosomes or released in the extracellular environment by the fusion with the plasma membrane [40]. The exosomes carry proteins that may be involved in their formation/maturation or also molecules that may be related to the metabolic activity of the organism in question. 

The microvesicles (MVs), in the other hand, have their formation directly from the release by direct budding of the plasma membrane, with dimensions ranging from 100 to 1000 nm, partially overlapping the size of the exosomes, and their distinct classification depends on their biogenesis mechanisms [41]. Therefore, the composition of the MVs is basically lipids similar to cellular membrane composition, cytoskeletal and other proteins with functions related to maintenance of the plasma membrane. However, to describe proteins components specific to MVs is a hard task, with variations in the literature. A considerable number of enzymes involved in the formation of aminophospholipids and translocase, flipases, and flopases are also present in the MVs, participating in the transfer of phospholipids into and out of the plasma membranes [41,42,43].

In recent years, EVs have been described as secretion mechanisms for a wide range of molecules to reach the extracellular environment in a large number of organisms [44,45,46,47,48]. Basically, their composition is responsible to mediate important roles in nutrition, physiopathogenesis and cell-to-cell communication. In fact, as cellular interactions can occur in a variety of ways [49], the release of EVs therefore compose one of the most important ways without cell-cell contact or indirect communication, along with processes through the secretion of soluble components [40]. 

In fungi, one of the hypotheses for the EVs release discussed for *Cryptococcus neoformans* is the accumulation of EVs between the membrane and the cell wall that can generate a directional pressure that forces the EVs to cross through a trans-cell wall trajectory; therefore the dimension of the released EVs would be modulated by the size of the wall pores. Another hypothesis discussed involves protein channels through the cell wall, which would promote the passage of EVs and potentially require cytoskeletal proteins that are commonly secreted in vesicles [50,51,52]. Alternatively, EVs’ secretion to the extracellular milieu is also involved in the remodeling of the cell wall by the action of degradation/synthesis enzymes. There is still much to be enlightened about the secretion of EVs, such as bio-molecular content and how the environment can in turn modulate all the contents present in the EVs.

The interaction process between microorganisms and their environment is closely related to the dynamics of microbial surface modifications, stimulated by nutrient acquisition, defense against other microorganisms or resistance to pressures of a host immune system. Thus, by contributing to the adaptation to various adverse conditions, the EVs released from the cell surface are conserved throughout the microbial life in bacteria, archaea, fungi and parasites [53].

## 3. Characterized EVs Throughout Other Kingdoms and Their Role in Microbial Pathogenesis

While much of the literature highlights the EVs associated with bacterial cells, currently EVs are considered key mediators, not only in the process of bacterial pathogenesis, but also of fungi and protozoa, making it an important research area to be explored. In gram-negative bacteria, the production of EVs occurs from the outer membrane (outer membrane vesicles, OMVs), as they become filled with periplasmic content. These OMVs allow bacteria to interact with their environment in several ways, increasing the chances of bacterial survival under stress conditions. These OMVs could mediate nutrient acquisition and protection as they regulate the density of interacting bacterial communities, such as biofilm formation, helping to provide structural support in multispecies environments. In addition, OMVs contribute to the release of virulence factors and modulation of the host immune system during pathogenesis and may confer resistance to antibiotics, constituting a mechanism of enhancement of bacterial adaptation and survival in the hostile host environment [54]. As they are directly involved in the virulence of bacteria, researchers have begun to explore the OMVs as a platform for bioengineering applications. Studies targeting the use of OMVs as a potential vaccine tool have been conducted and are currently in clinical trials [55,56,57,58].

Structurally, gram-negative bacteria differ from gram-positive bacteria. These have no outer membrane and are surrounded by a layer of peptidoglycan [59]. Thus, the EVs released by gram-positive bacteria have different biogenesis and content of gram-negative EVs. Pathogenic gram-positive bacteria, such as *Staphylococcus aureus*, *Mycobacterium ulcerans* and *Bacillus* spp. are known to release EVs. Some studies investigating the EVs content of gram-positive bacteria, pointed to the presence of enzymes involved in their own release to the extracellular milieu, such as hydrolases and peptidoglycan-degrading enzymes and toxins. A work carried out with *Bacillus anthracis* indicates that the bacteria secretion of toxins is EVs-associated, as they concentrate the release of these toxin components into these structures, increasing the toxin damaging potential to the target cells [60]. Therefore, gram-positive bacteria EVs have gained attention for emerging as important components for the design of vaccines and as targets for passive immunization strategies. Rivera and colleagues have shown that *B. anthracis* EVs containing toxin induced robust immune responses in BALB/c mice upon vaccination, which led to higher survival rates in animals challenged with the bacteria [44,60]. Similar results were obtained in studies with *Mycobacterium tuberculosis* EVs. Mice immunized with mycobacterial EVs induced strong T-helper type 1 cell responses (Th1), elicited antibody production, and reduced bacterial burden [44,61].

Several studies suggest the involvement of EVs in fungal pathogenesis, as transporters of virulence factors to the extracellular environment [44,62,63,64,65,66]. Glucosylceramide (GlcCer), an important virulence factor of the fungus *Cryptococcus neoformans*, is present in the EVs released by this fungus [67]. Fungal EVs are also associated with the storage and export of fungal pigments, such as melanin. Studies with purified EVs of *C. neoformans* show the presence of dense and dark granules and the ability of melanin biosynthesis in the presence of L-3,4-dihydroxyphenylalanine (L-DOPA), with subsequent transport of this polymer to the cell wall [68]. In addition, small RNA molecules are found composing fungal EVs and their involvement in the communication between cells, including communication and modulation of host cells, is suggested as a possible attempt to make it more susceptible to infection [69]. As well as being associated with carriers and release of fungal virulence factors, EVs play an important role in sugar metabolism, cell wall architecture, cell signaling, lipid metabolism, and cell growth/ division [44].

## 4. Protozoa EVs and Pathogenesis

Production of EVs appears to be a common mechanism for secretion among parasites as several studies have described the production of these structures by the protozoa *Plasmodium* sp. [40,70], *Leishmania donovani*, *L. major* and *L. mexicana* [71]; *Trypanosoma brucei* [72,73], the amoebas *Dictyostelium discoideum* [74,75] and *Acanthamoeba castellanii* [39] and in *Trematodes* such as *Echinostoma caproni* and *Fasciola hepatica* [76]. The EVs released by protozoa consist of important processes in interaction with the host, containing crucial molecules for the establishment of infection, unlike previous beliefs that considered them as a waste of energy and escape valve of metabolism [77]. The vesicles play a key role in the pathogenesis mediated by *Leishmania* spp. and *Trypanosoma cruzi*. They communicate with cells of the host immune system, are involved in inflammatory processes and protein export pathways. Some of the publications have also demonstrated the importance of these exosomes as a therapeutic target potential for infectious diseases [70,78].

In *L. donovani*, the release of EVs through the shedding of these structures on the surface of the parasite’s body was observed through scanning electron microscopy. Proteomic analysis of *Leishmania* sp. secretome was able to identify 358 proteins, many that were present within the released exosomes [79]. Exosomes containing proteins of *Leishmania* were identified within the cytoplasm of infected macrophages, suggesting that EVs participate in the pathogen-host interaction [71,80], working as important mediators involved in the production of interleukin-8 (IL-8). Therefore, there is a potential of involvement of EVs in the increasing of the parasite’s virulence.

EVs secretion was also characterized in trypomastigotes of *Trypanosoma cruzi*. These EVs carry different molecules, such as the glycoprotein gp85, a trans-sialidase superfamily, and α-galactosyl glycoconjugates [42]. Proteomic analyzes have further identified the presence of a large numbers of proteins containing nucleic acid binding sites and ribosomal proteins. Additional analysis also displayed contents of distinct types of small RNAs within protozoa EVs, which could have their packing and released into EVs modulated by nutritional stress and may play a role in the parasite-host interaction [81]. Trocolli-Torrecilhas and colleagues showed that pretreatment of mice with *T. cruzi* EVs, following challenges with parasites resulted in higher animal mortality rates than EVs untreated animals, developing severe cardiac pathologies with intense inflammatory reaction, demonstrating that vesicles may also be a key component in the pathogenesis of *T. cruzi* [42]. A considerable increase in IL-10 and IL-4 production has also been observed, which leads to the conclusion that EVs have immunomodulatory potential and increased parasite load. These data suggested that *T. cruzi* EVs could facilitate parasite dissemination and pathogenic mechanisms [42].

## 5. Recently Characterized EVs of FLAs and Their Impact in Pathogenesis

Initial studies in *Dictyostelium discoideum* model raised the hypothesis of the participation of EVs released by protozoa in cell-to-cell communication [75,82,83]. The production of “nanovesicles” in *D. discoideum*, with diameters ranging between 50 and 150 nm, was described; however, these EVs were later reclassified as exosomes based on their sizes [40,41,74]. In the same work, the presence of actin, proteins involved in actin metabolism, ribosomal proteins, a galactose binding lectin, mitochondrial proteins and RAS proteins were described by proteomic analysis. These proteins were characterized as important extracellular content to allow the interaction and communication between cells in order to determine their differentiation. For instance, EVs in the conditioned medium could be responsible for changing the fate of *D. discoideum*, by inducing cell aggregation and "social" apoptotic death mediated by mitochondria [40,74]. The molecules, including proteins, peptides, amino acids, nucleic acids, steroids, and polyketides, are used as intercellular mediators among the trophozoites, as well as in signaling and interference to plants and animals [83]. 

The biogenesis and the mechanisms involved in EVs secretion in *Acanthamoeba* sp. remain unknown. However, our group performed a descriptive study on the molecular components secreted by *A. castellanii* under different growth conditions and the production of EVs [39], whose composition was similar to EVs of distinct microbes. As observed, the EVs of *A. castellanii* had their size modulated according to the environment that the trophozoites inhabitates, as well as the total number of secreted proteins; *A. castellanii* grown in rich media secreted high levels of ribosomal proteins, proteins related to locomotion, and signaling pathways, corroborating with the hypothesis of cell-to-cell communication involvement of *D. discoideum* EVs [75,82,83]. 

Nutritional stress promoted changes in the content of EVs secreted by amoebas; in the cultivation condition named, by the authors as “stress condition", in the absence of glucose, additional 26 proteins were observed when compared to the cells in homeostasis. 

However, the adaptation of *A. castellanii* to nutritional stress resulted in the secretion of EVs containing proteins related to protein and amino acid metabolism, cellular stress, and oxidative metabolism [39]. Additionally, the growth of amoebae under nutritional stress resulted in plasticity of the carbohydrate metabolism, which enables them to use different resources to be successful during the process of colonization of different niches [39]. The findings confirmed the presence of proteins related to the metabolism of sugars in the soluble secretome free of EVs, indicating the involvement of this content in extracellular digestion and nutrient acquisition [39].

The EVs released by these protozoa are rich in proteases. These enzymes play important roles in the pathogenesis of infectious diseases and cancer [44,45,47,48]. In a comparison to other amoebas, one of the species mostly characterized regarding the production of proteases is *Entamoeba histolytica*, whose cysteine proteases have implications in the degradation of the protective barrier of the intestinal mucosa, promoting invasion of the pathogen in the host [84,85].

In *A. castellanii*, proteases consist of important virulence factors and are highly expressed in pathogenic strains. This class of proteins also plays important roles both in pathogenicity and in differentiation of amoebae between cyst/trophozoite [86,87]. The T4 genotype of *A. castellanii*, associated to fatal brain disease, GAE and sight-threatening keratitis, secretes mainly serine proteases, whereas other genotypes are able to produce metallo and cysteine proteases [87]. Proteases were remarkably abundant in the EVs produced by this organism, which could be directly implicated in host tissue damage and host cell death.

Thus, besides the presence of proteases, other classes such as kinases and glycosidases were also found in EVs of *A. castellanii* and could fulfill the perfect mechanism for cellular signaling and addressing enzymes capable of digesting extracellular matrix and even escape from the host’s immune system [88,89]. Thus, this enable the parasite to colonize host tissues, and at last determine the tropism of *A. castellanii* to retinal tissues, resulting in ocular keratitis or the central nervous system, resulting in encephalitis [89]. 

Some markers in the protein content of *A. castellanii* EVs could be identified when comparing to those previously published in other microorganisms [39], such as *T. cruzi* [90], *L. major* [79], and *Plasmodium* sp. [91]. These markers include actin (L8HCZ7) (www.uniprot.org entry), as found in *T. cruzi*, *L. major* and *Plasmodium* sp.), heat shock proteins hsp83 (L8H6T6, as found in *T. cruzi* and *L. donovani*) and Hsp60 (L8GUV2 as found in *T. cruzi*), histone 2B (L8H4F8 as found in *L. major* and *Plasmodium* sp.) and serine proteases (L8H1H6 and L8HEC6 as found in *T. cruzi*).

**Figure 2:**
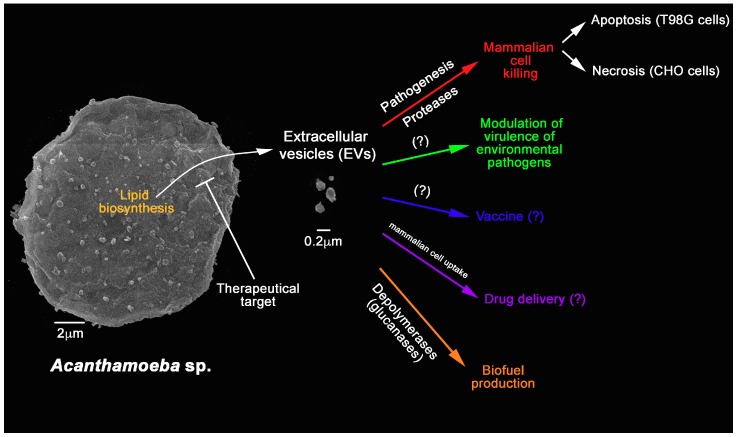
Overview of the characterized functions of *A. castellanii* EVs and their potential applications in bioengineering and biomedical sciences. *A. castellanii* EVs’ synthesis could be target for therapy. They are also known to participate in the pathogenesis of *A. castellanii* by killing mammalian cells. There is a growing consensus that they might also be able to alter the virulence of environmental interacting pathogens. Further applications include the use as vaccines, drug delivery platforms, and as sources of depolymerases that could be used in the biofuel production.

EVs released by *A. castellanii* and *D. discoideum* were uptaken by mammalian cell lines. Upon interaction, high amounts of *A. castellanii* EVs accumulated inside the cytoplasm of epithelial (CHO) and blood-brain barrier cells (T98G cells) in a time-dependent fashion, and finally localized around the nucleus [39]. As similar to *A. castellanii,* accumulation of vesicles of *D. discoideum* [74] in HeLa cells occurred within 30 minutes incubation. Further incubations of *A. castellanii* EVs could destabilize these epithelial and blood-brain barrier cells, inducing necrotic or apoptotic cell death, respectively (Figure 2) [39]. Therefore, the presence of EVs of potentially pathogenic FLAs may modulate the entire process of the pathogenesis of these agents in mammalian hosts. 

Besides *A. castellanii*, the role of EVs in the process of pathogenesis in protozoa and other microorganisms has been described in some species extensively [44,45,47,48,62,63,66,70,73,78,92,93]; however, it is necessary to have additional understanding of these EVs functions in both pathogenesis and in the ecological regulation that these molecules can exert between cells or in the microbial community, such as in a biofilm (Figure 2). As for the biogenesis of these exosomes in *A. castellanii*, their mechanisms have not been elucidated yet, requiring further investigation.

### Lipids in A. castellanii EVs

The first analysis of the lipid components of *A. castellanii* dated from 1969, with the description of total lipids of trophozoite as 52% of neutral lipids and 48% of polar lipids. Triglycerides represented 75% and free sterols 17% of neutral lipids. Regarding the identified phospholipids, phosphatidylcholine, phosphatidylethanolamine, phosphatidylserine, phosphoinositol, and diphosphatidylglycerol consisted of the main classes. Phosphoinositol is unique in the fact that it contains 1:4:0.5:1.1 (fatty acids:aldehyde:inositol and phosphate, respectively), without the presence of glycerol. No sphingomyelin, cerebrosides, psychosin, nor glycoglycerides were detected; instead, the authors detected small amounts of unidentified long chain sugars and bases [43], which were further characterized by the authors. 

A couple of years later, the same group [94] specifically characterized the lipidic constitution of *A. castellanii* plasma and phagosome membranes isolated by low-speed/sucrose gradient centrifugation. The isolated membranes and phagosomes displayed a very high sterol to lipid ratio (0.98 moles/mole) and very low concentrations of lipid inositol and glycerides. Comparing the membrane composition to the whole cells, the phospholipids consisted of more phosphatidylethanolamine and phosphatidylserine and less phosphatidylcholine. No differences in the ratio of total saturated/unsaturated fatty acids between the plasma membranes and whole cells were noticed, despite the punctual differences in fatty acid compositions of corresponding phospholipids. 

The previous unidentified long chain sugars and bases composed a macromolecule, which was isolated from the whole cell and indistinguishable from the component of the amoeba plasma membrane, also containing a non-phospholipid phosphorus [95]. These lipophosphonoglycan (LPG), identified as a major component of the *A. castellanii* plasma membrane on both sides [96], contained inositol (8%) and anteiso-C24 and C25-phytosphingosines (13%), in addition to the previous identified neutral and amino sugars (26 and 3% respectively) [97,98]. In fact, upon exhaustive delipidation and butanol extraction, anteiso-C24 to anteiso-C28 chains were detected, but anteiso-C25 phytosphingosine was the most abundant, constituting more than 50 % of all LPG [99]. Like in *Leishmania* sp. [100], these *A. castellanii* LPG could work as *Acanthamoeba*-associated molecular patterns for the stimulation of the Toll-like receptors expressed by innate immunity cells [101].

Gonçalves et al [39] also performed, for the first time, the lipid analysis in *A. castellanii* EVs and observed that the composition resembles the lipid proportion in trophozoites. In the lipid composition of the purified EVs of both systems, homeostasis and stress, the following lipids such as diacylglycerol; monoacylglycerol; phospholipids; free sterol; free fatty acids; and esterified cholesterol were identified. In order to evaluate the existing neutral lipids, the EVs were submitted Gas-chromatography coupled to mass spectrometry (GC-MS), which identified cholesterol, ergosterol and stigmasta-5,7,22-trien-3α-ol_(7-stigmasterol); the last being described by the first in *A. castellanii*, whereas cholesterol and ergosterol had previously been described in the plasma and phagosome membranes of *A. castellanii* (as mentioned above [94]) and the related species *A. polyphaga* [102]. Ergosterol is a typical sterol isolated from fungal cell membranes, whereas stigmasterol is commonly found in plant roots and algae [103]; both sterol have also been described in total extracts of *Acanthamoeba polyphaga* [102]. In a more recent study, different lipid precursors from the ergosterol synthesis pathway have been described as the main component of environmental (Neff) and pathogenic (T4) strains; however, cholesterol, demosterol, campesterol, stigmasterol, or 7-dehydrostigmasterol was not detected. With the evidence of ergosterol synthesis in *A. castellanii*, it becomes possible to use antifungals such as azoles and amphotericin B as a potential therapeutic options to infections caused by FLAs (Figure 2) [104].

As fatty acid of 14 to 22 carbons chains were also identified in EVs of *A. castellanii*, the presence of long-chain carbon fatty acids lipids may consist also in a potential target for drug development or therapeutic features for *A. castellanii*, since similar molecules have not been described in mammals, bacteria or fungi (Figure 2). Another possible function of long chain fatty acids, described in *A. castellanii,* could be related to some step in membrane remodeling, as previously described for *A. polyphaga* [105].

## 6. Further Potential Applications of FLAs EVs

The application of EVs on the production of vaccines has been the subject of much attention by several studies. EVs usually offer a more stable conformational condition for the protein components, are able to circulate intact in body fluids and associates efficiently with antigen-presenting cells (Figure 2) [47]. For instance, dendritic cells loaded with tumor derived exosomes have demonstrated activity in the control of tumors for cancer immunotherapy and have been the first studies performed regarding EVs as vaccine tools [106]. Currently, vaccines from bacterial EVs, such as the Meningococcal group B vaccine (Bexsero), which showed high effectiveness in regions of the outbreak, are under development. Several studies focusing on the immunobiological activities of fungal EVs have also been carried out. EVs of *C. neoformans* are biologically active *in vitro*: They stimulate nitric oxide, cytokine (TNF-α, IL-10, and growth factor [TGF-β]), and fungicidal activity in macrophages [46]. EVs of *Paracoccidioides brasiliensis* also demonstrated a role in cytokine stimulation by macrophages [107]. However, as fungal EVs interfere in both fungal virulence and host stimulation, it is not entirely clear how this protective mechanism would occur; therefore, no fungal vaccines have been characterized. Interestingly, many studies have been developed using EVs of non-pathogenic fungi, as potent stimulators of the host immune response, and consequently, protection against fungal infections. 

Studies on mammalian cell-derived exosomes and bacterially derived EVs for the development as vaccines for immunotherapy are increasing. However, EVs of microbial origin could induce a potentially strong immunogenicity, which may be a potential drawback for their in vivo use. In contrast, many proteins present in cell-derived exosomes are not detected in *D. discoidium* nanovesicles. This could minimize the risk of undesirable immune responses upon in vivo administration, such as those triggered by tumor-released exosomes. A first in vivo study upon the immunogenicity of *D. discoidium* nanovesicles, intravenously injected in BALBc mice, has shown a specific antibody response, but no pyrogenic response, nor any inflammation was detected by cytokine evaluation. Thus, to explore key models such as *D. discoideum* as bio-engineering designer able to formulate vesicular drug carriers becomes a field of important application. 

Several reports also describe the usage of EVs of *D. discoidium* as platforms for drug delivery, as these structures easily get to the mammalian intracellular milieu [74,75,108]. Previous studies have demonstrated that *D. discoidium* cells grown in the presence of exogenous molecules, such as a DNA specific dye, for example, produced dye-loaded nanovesicles as a detoxification mechanism [75]. In addition, it is shown that these nanovesicles are able to transfer their contents to the nuclei of naive *Dictyostelium* or human leukeamia K562r cells, which are resistant to vital labeling of their nuclei by the dye.

These nanovesicles of biological origin could also be loaded with therapeutic molecules and then used as a nanodevice for cellular drug internalization [74,108]. Lavialle et al. explored the potential use of *D. discoideum* released nanovesicles as in vitro drug carriers for cancer therapy. Nanovesicles filled with hypericin, a fluorescent therapeutic photosensitizer assayed for antitumoral photodynamic therapy, efficiently delivered the drug as the fluorescence signal was located exclusively in the perinuclear area of the skin fibroblast (HS68) and cervical carcinoma (HeLa) human cell lines [74]. In the other hand, as efficient drug deliver tools, these secreted nanovesicles could also act as detoxifiers, carrying drugs out of cells, and therefore acting as “Trojan horses” by being capable of transferring these drugs among parasite cells, but also to human cells [74,75,108]. 

## 7. Conclusion Remarks

Based on these aforementioned concepts and the biochemical similarity to *D. discoidium* EVs, *A. castellanii* EVs could work efficiently as liposome carriers of immunogenic proteins, being able to activate the immune system, inducing a protective vaccination response [108] (Figure 2). The same line of reasoning can be proposed for *A. castellanii* EVs as efficient drug delivery system, since these EVs could also easily access the cytoplasm of mammalian cells in a more eficiente manner than the characterized *D. discoidium* EVs [39], for the efficient delivery if their intact contents (Figure 2).

*Acanthamoeba* are professional phagocytes and are able to secrete a wide variety of enzymes to digest the engulfed particles, and further use them as nutritional source for energy acquisition. As EVs from *A. castellanii* are efficient carriers of several glycosidase, lipases and proteases, their biological activity and potential as depolymerizing agents could be further explored [39,109]. Glycosidases such as glycosyl hydrolases, beta-glucosidases, galactosidases and mannosidases, xylosidases and alpha amylases were abundant in *A. castellanii* EVs. These compose a universe of carbohydrate-processing enzymes that have had wide industrial applications due to their hydrolase efficiency, such as in the food and bio-bleaching in the paper and pulp industry [110,111,112]. As the glycosydases are also involved in many biological processes such as cell growth, cell recognition and parasitic infections, they have become attractive targets for the pharmaceutical industry [113]. These *A. castellanii* EVs could have additional applications in the biotechnology, such as in the boosting of biofuel industry, by their potential of solid biomass degradation and their conversion into liquid biofuels (Figure 2).
